# Lessons learnt during the process of setup and implementation of the voucher scheme in Eastern Uganda: a mixed methods study

**DOI:** 10.1186/s13012-015-0292-3

**Published:** 2015-08-06

**Authors:** John Bua, Ligia Paina, Elizabeth Ekirapa Kiracho

**Affiliations:** Department of Health Policy Planning and Management, School of Public Health, Makerere University College of Health Sciences, P.O. Box 7072, Kampala, Uganda; Department of International Health, Johns Hopkins University Bloomberg School of Public Health, 615 N. Wolfe Street, Baltimore, MD 21205 USA

**Keywords:** Vouchers, Maternal health, Health financing, Health system strengthening, Uganda, Africa

## Abstract

**Background:**

In spite of the investments made by the Ugandan Government, the utilisation of maternal health services has remained low, resulting in a high maternal mortality (438 maternal deaths per 100,000 live births). Aiming to reduce poor women’s constraints to the utilisation of services, an intervention consisting of a voucher scheme and health system strengthening was implemented. This paper presents the lessons learnt during the setup and implementation of the intervention in Eastern Uganda, in order to inform the design and scale up of similar future interventions.

**Methods:**

The key lessons were synthesised from a variety of project reports, as well as qualitative data drawn from six focus group discussions and four in-depth interviews conducted in the Buyende and Pallisa districts during the implementation phase of the voucher scheme.

**Results and Conclusions:**

To promote the successful implementation of interventions with demand and supply side initiatives, such as voucher schemes, the health system should be able to respond to the demand created by providing the additional required resources such as health workers, essential supplies and equipment. Involving a diverse, multi-sectoral group of stakeholders is important for addressing the different barriers experienced by women when seeking maternal health services. Voucher schemes should have a mechanism of detecting unintended consequences and mitigating them. Sustainability plans should be built into such interventions to maintain the gains achieved. Lastly, health policy planners can use this information to develop follow-up programmes to test modified versions that are more sustainable. Such programmes could use locally existing community structures for management and resource mobilisation for self-sustainment.

## Background

Although Uganda has achieved reductions in maternal mortality over the last decade [1], the current maternal mortality rate of 438 per 100,000 live births remains unacceptably high. The underutilisation of maternal care services like skilled delivery is one of the factors contributing to that high mortality [1]. The underutilisation of maternal care services is due to a combination of demand and supply side problems. The demand side problems include long distances to facilities, lack of transport, unaffordable transport costs, lack of power to make decisions among women, preference for traditional delivery positions and lack of knowledge [2–6]. The supply side barriers include unaffordable care costs, lack of skilled care, drug stock outs, lack of equipment, poor health worker attitudes towards clients and late referrals [2, 5, 7, 8].

Over the years, the government invested in supply side interventions, such as construction of health facilities, recruiting trained personnel, supplying drugs and medical equipment [9, 10]. Despite such efforts, the utilisation of maternal services has persistently remained low. Less than half (48 %) of pregnant women attend the fourth antenatal care visit and not more than two thirds of these women (57 %) deliver in health facilities [1]. The quality of care in public facilities has remained unsatisfactory with frequent shortages of equipment, supplies and drugs [11, 12]. Supply side incentives provide little inducement to encourage patients, especially those from vulnerable communities, to utilise facility-based services. They also do not give service providers an incentive to provide better services beyond the basic essentials. However, evidence shows that combining demand and supply side incentives takes advantage of each to intensely improve utilisation and quality of care services [13].

In an attempt to address the above constraints, Makerere University School of Public Health, in collaboration with Johns Hopkins University, with funding from the Melinda Gates Foundation and United Kingdom Department for International Development (DFID), piloted the Safe deliveries project. The project applied a voucher scheme with both demand and supply side initiatives.

The demand side initiative included two vouchers, for transport and maternal care services. A transport voucher was provided to pregnant women and recently delivered mothers from poor communities. The transport voucher could be used with local transport providers based in the communities where the beneficiaries resided. The services voucher covered costs for antenatal, delivery and postnatal care. These demand side incentives subsidised the costs incurred during the process of seeking maternal care services thus providing protection from catastrophic expenditure that could have occurred when getting treatment for medical emergencies [14]. Demand side incentives also provided pregnant mothers a chance to choose which provider to seek services from and therefore encourage health providers to provide quality care through increased competition [15]. The supply side incentives served as a complement to the demand side in order to improve the quality of care services. The supply side incentives provided by the project included training health workers how to conduct Safe deliveries project, provision of basic essential equipment, drugs and support supervision.

During the implementation period, the intervention evolved in response to changes in the political and economic context, as well as to stakeholders’ needs. As a result, the implementation of the intervention did not always occur according to plan because of several anticipated and unanticipated factors that influenced its delivery. Thus, subsequent outcomes were both positive and negative. Systematically documenting these factors can provide lessons about the process of implementation and adaptations which can inform the replication and successful scale up of similar interventions in different settings. Despite the value of documenting adaptation, this is rarely done in practice or published in the peer-reviewed literature. This paper contributes to filling this significant gap in the implementation research literature. We provide a brief description of how the Safe deliveries project was setup. We then draw attention to the lessons learnt during the process of project setup and implementation.

### The Safe deliveries project study

The Safe deliveries project was implemented for 2 years (2009 to 2011) in four districts: Buyende, Kamuli, Pallisa and Kibuku districts of Eastern Uganda [16]. The area has an estimated population of 1,293,990 with a large proportion (25 %) earning less than a dollar a day, through subsistence farming [17].

Initially, this project was supposed to be implemented in four health sub-districts (HSD) of Kamuli and Pallisa districts. Each district was supposed to have an intervention and a control HSD. However, after the study was designed and initiated, the Ugandan government enacted another district-splitting effort which resulted in Kamuli being split into Kamuli and Buyende districts. Then, Pallisa was split into Pallisa and Kibuku districts. Buyende and Pallisa districts were the intervention areas while Kamuli and Kibuku districts were used for comparison. The criteria used to select the study districts included having health facilities and health workers that can offer emergency obstetric care. There were 104 health facilities from the public, private not for profit, and private sectors that participated in the intervention (Table 1). The facilities were selected by the district health departments based on the standard requirements from the Ministry of Health.

**Table 1:** The number and type of health facilities in the study area

		Govt.	NGO	Private	Total
Buyende	Hospital	0	0	0	0
	HC IV	1	0	0	1
	HC III	4	2	0	6
	HC II	3	4	0	7
	Total	8	6	0	14
Kamuli	Hospital	1	1	0	2
	HC IV	2	0	0	2
	HC III	11	0	0	11
	HC II	17	17	2	36
	Total	31	18	2	51
Pallisa	Hospital	1	1	0	2
	HC IV	2	0	0	2
	HC III	10	2	0	12
	HC II	7	2	0	9
	Total	20	5	0	25
Kibuku	Hospital	0	0	0	0
	HC IV	1	0	0	1
	HC III	7	2	0	9
	HC II	3	1	0	4
	Total	11	3	0	14

The project was first piloted in 14 health units in Buyende district for 3 months and 2 Kamuli district hospitals. It was then scaled up and implemented in both Buyende and Pallisa districts for a 1-year period. In Pallisa district, it worked with nine health facilities.

The beneficiaries were identified using universal targeting in the intervention areas. Therefore, all the pregnant women residing in the intervention area were entitled to vouchers. The distribution of vouchers was done by the health workers during antenatal care, with the assumption that over 90 % of pregnant women in Uganda attend at least one antenatal care session at the health facility [1].

The health care providers were reimbursed after submitting evidence of providing service to the beneficiaries. The payments were delivered in cash to the health facilities. The providers in private not for profit and private for profit health facilities were reimbursed the full cost of their services. The public providers received 75 % of their costs from the project because they received more funding from the government (Table 2). These funds were meant to be used by providers to improve the quality of services through ensuring adequate medical supplies, repair of facilities and facilitating support staff.

**Table 2:** The voucher value for health care services and transport

	Health care service	Value in private facility (US$)	Value in public facility (US$)
Pilot phase	ANC 1	0.96	0.72
	ANC 2, 3 and4	1.15	0.86
	Delivery	5.76	4.32
	Caesarean section	57.58	43.19
	PNC	1.15	0.86
	Transport service	Transport value (US$)	
	To health facility	2.5	
	Referral to district hospital	7.5 to 10	
Implementation phase	ANC 2, 3 and 4	0.46	0.32
	Delivery	3.07	2.3
	Caesarean section	49.9	24.95
	PNC	0.46	0.32
	Transport service	Transport value (US$)	
	To health facility	1.5 to 2.5	
	Referral to district hospital	7.5 to 10	

During the pilot phase, the voucher for care services covered four antenatal visits, facility delivery, emergency obstetric care, such as caesarean sections at district hospitals, and one postnatal visit within the first 7 days. The transport voucher covered transport costs to and from the health facility during the visits. The transport voucher also covered referral costs from the lower level health facility to the district hospital for emergency care.

During the pilot phase, the transport voucher was valued at a flat rate of US$2.5 per trip within the intervention area. This was based on negotiations with the transport providers. However, the response overwhelmed the scheme resulting in high voucher costs. Consequently, the benefit packages were revised. The transport voucher value was revised based on the distance from the beneficiaries’ village to the health facility. The revised transport voucher value ranged from US$ 1.5 to US$ 2.5 per trip.

During the implementation phase, the voucher for maternal care services covered facility delivery, one postnatal visit, sick newborns and caesarean sections. However, the pregnant women enrolled during the pilot phase continued receiving antenatal services during the implementation phase.

Sensitization was also done within the communities to create awareness about the intervention. The methods used included district level meetings, posters, mobile film vans, radio talk shows and radio messages about maternal care in local languages. Further details about the design of the Safe deliveries project are provided in a paper by Ekirapa-Kiracho et al. [16].

### Methodology

Two sources of secondary data were used for this study. First, a review of study documents that included research team field reports, study report and the project implementation manual during the pilot and implementation phase of the study was conducted in order to document the process of how key intervention elements changed over time.

Second, transcripts from focus group discussions and in-depth interviews that were done with study participants and stakeholders were analysed to explain the adaptations that occurred.

The focus group discussions were done with women, men and transporters while the in-depth interviews were done with community opinion leaders. These leaders included male village council representatives from two villages in Buyende, a female local council representative from a sub-county in Pallisa district and a transporters’ representative from Buyende district. The focus group discussions were conducted between 28 September and 1 October 2010 while in-depth interviews were conducted between 6 and 16 December 2010. The women interviewed were beneficiaries of the programme. The men interviewed were a mix of those whose spouses benefited from the vouchers and those whose spouses did not. The transporters interviewed were directly involved in the programme because they provided transport services to pregnant women.

The key lessons were then synthesised from the project reports, field reports made by the project team members and district health team during the implementation and qualitative data drawn from the above interviews. The qualitative data was analysed using thematic analysis which involved extensive reading of transcripts, coding of the data and categorisation into themes. Table 3 below summarises the data sources we selected to identify the lessons learned during the implementation of the Safe deliveries project voucher scheme.

**Table 3:** Data collection matrix

Methods/source of data	Study population	Sample size	Lessons
FGDs	• Mothers who used the vouchers	• 2 FGDs of 10 mothers each who used the vouchers in Buyende district	• Benefits of working with local private providers
	• Men whose spouses used vouchers		
	• Transporters	• 1 FGD of 15 mothers who used the vouchers in Pallisa district	• How to identify the right incentives for providers
			• Unintended consequences
		• 1FGD of 10 men in Buyende district	
		• 1 FGD of 11 transporters in Pallisa District	
		• 1 FGDs of 12 transporters in Buyende district	
IDIs	• Community leaders	• 1 IDI of male council representative from Iringa village in Buyende district	• Engaging community
			• How to target beneficiaries
		• 1 IDI of female council representative from Kamuge Sub-County in Pallisa district	• Benefits of working with local private providers Sustaining the intervention
	• Transporters representative	• 1 IDI of male council presentative from Wesunire village in Buyende district	
		• 1 IDI of transporters’ representative from Kidera Sub-County in Buyende district	
Desk review	• Safe deliveries project report		• District response to increased demand
	• Safe deliveries project/field notes		• Verification using registers
	• Safe deliveries project implementation manual		• How to target beneficiaries

### Ethical considerations

Approval to conduct this study was sought from the Makerere University Higher degrees Research and Ethics Review Board and the National Council of Science and Technology. Informed consent was obtained from the study participants before they were interviewed.

## Results and discussion

A number of lessons related to the implementation process of the voucher scheme were identified and organised into key themes.

### Theme 1: Engaging community and service providers to foster buy in for the voucher scheme

In keeping with previous work [18], one of the lessons learnt was that it is important to raise awareness about the scheme. In areas where this was not well done initially, uptake of the scheme was poor. The use of local community leaders to mobilise community support and collaboration for implementing the scheme was an effective way of engaging with the community. This was because local leaders are trusted by their communities which were more inclined to listen and trust messages from them. One of the local leaders commented that:

When you sensitize women on the scheme ............, involve community leaders because the people listen more to their leaders or elders. (In depth interview with female council representative from Kamuge Sub-County, Pallisa district during the implementation phase)

Feedback from the stakeholders revealed that the radio talk shows or radio messages were more effective in raising awareness if they are aired at regular times. This encouraged the listeners to tune in for the programme.

Another lesson learnt was that it is important to maintain continuous dialogue with the service providers. Constant communication with the transporters was achieved through continuous dialogue meetings and a phone line for open communication. For the health workers, regular stakeholder meetings were held and support supervision visits were conducted when delivering cash payments. This open communication allowed the development of trust between the providers and the programme implementers. These channels of communication motivated the providers because they provided a venue for sharing their concerns. It also made them own the intervention and encouraged innovative mechanisms to share best practises that could lead to improvements in access to transport and maternal health services. Lastly, it provided an opportunity to communicate and resolve problems amicably. For example, when there were delays of payment, the providers agreed to wait without disrupting the programme.

### Theme 2: District Health system response to the increased demand for services

As mentioned in the introduction, the demand for services generated was higher than expected and created new challenges to the existing ones especially during the pilot period [16]. A local transport provider observed that:

The problem we find is that we wait for long hours; we have few health workers in the health facilities and there are big numbers of women coming to get maternal health services. So we request you to address this problem so that it can be solved. (FGD for transporters, Kidera village, Buyende district)

This demonstrated the fact that when demand is generated, the health system needs to be able to respond by increasing the supply of services. When the patient numbers increased, the number of district health staff remained the same in some facilities. Therefore, the few existing health workers became overstretched. This resulted in long waiting times at health facilities. The government ban on recruitment of new health workers due to lack of funds to pay their salaries at the time meant that districts could not respond immediately to the staff shortages.

The increased demand also led to increased utilisation of drugs and medical sundries. Despite the supplies from the National Medical Stores and additional supplies provided by the voucher scheme, stock outs of essential drugs were observed. A mother who benefited from the transport intervention noted that:

They can bring drugs today and when you come the next day you don’t find anything. They just write for you medicine to buy from private drug shops. (FGD for women in Nkondo village, Buyende district)

When mothers came to the health units and waited for very long, or did not receive services or drugs, they became discouraged. This in some cases may have led to the shunning of health facilities.

Lastly, the existing infrastructure was also strained due to lack of adequate maternity space and delivery beds in the lower level facilities. Construction of new buildings is a capital investment which could not be met from the voucher funds. As a result of this limited space, in some cases, the mothers could not stay at the facility for more than 24 h after giving birth as recommended.

### Theme 3: How to target the beneficiaries

A universal distribution system was used to target all pregnant women located within the intervention areas of these rural districts since they were all considered poor. It was difficult to distinguish the very poor from the well-off because this would have required the study to develop its own ranking system, an endeavour that would have called for additional financial resources which had not been budgeted for. Although this method was easy to implement, it may have contributed to some wastage of resources since both the very poor and the least poor benefited. Distribution of the vouchers at health facilities was beneficial because it encouraged more women to attend ANC services but it increased the workload for the health workers. Furthermore, it could have led to the exclusion of women who were not able to come to the facility. However, in this programme, such women could still receive vouchers at the time of delivery so this reduced their possible exclusion.

### Theme 4: Identifying the right incentives for providers

The programme also showed that in a voucher scheme like this one, it is not only the type of incentive that matters but also the amount of the incentive. It was necessary to keep negotiating with providers, health workers and transporters to ensure that the programme provides the right amount of incentives. Failure to do this could have led to minimised effects of the incentives. Similar findings were reported by Sengooba [19]. Transport costs using a motorcycle was US$2.5 during the pilot irrespective of the distance covered. However, during the implementation phase, this rate was reduced to between US$1.5 and US$2.5. During the pilot phase, the transporters were very active, and they travelled even to villages that were very far to search for the mothers. However, when the rates were revised, the transporters preferred to transport pregnant women who were nearer to the health facility, neglecting those who stayed far in remote villages. This suggests that sometimes private for profit partners are driven by profit, so attention should be paid to this when negotiating prices with private for profit partners. The prices for the health facility vouchers were also changed twice. The initial change after the pilot resulted in lowering of prices; thereafter, feedback from the health workers after a few months of implementation indicated that the amount allocated was not enough for achieving the intended benefits (money for allowances, supplies and equipment). In response, the amount was increased to achieve the intended benefits.

### Theme 5: Benefits of working with local private providers

Such interventions bring to light other partners who can contribute to improving utilisation of maternal and child health care services; for example, the transporters became agents of change. The transporters moved from household to household in villages searching for pregnant women, who they encouraged to seek care at the health facilities. This was because the women with vouchers became potential clients for the transporters. This trend was confirmed by one of the community leaders below:

In fact the transporters used to search for these women from the villages and bring them to the health centres. (In depth interview of male council representative in Iringa village, Buyende district)

### Theme 6: Challenges with voucher verification using facility registers

The verification team used health facility registers to verify both transport and MCH service vouchers presented for payment. However, community individuals conniving with some health workers and transporters tried to take advantage of the voucher system through distributing forged vouchers, inflating registers, not recording in registers and reclaiming unused vouchers from women who delivered at home. This finding was similar with the literature which acknowledges fraud as one of the problems encountered by voucher schemes [20–22]. Therefore, the verification team selected a number of women randomly from facility registers and conducted household visits in villages to follow up women who had benefited from the vouchers. The team discovered that maiden names used by some pregnant women in the health facility register were not known by the locals in the villages. Instead, they were known by their husbands’ first names which were not recorded in the facility registers. This made the follow-up exercise difficult for some cases, underlining the difficulty of tracking patients in areas where there is no active national system for identification.

This requires the need to have a strong system of detecting fraud and confirming provision of services.

In this study, the unused vouchers could not be easily traced. A clear way of tracking vouchers distributed and those used is also important to avoid having excess vouchers that could be misused. Household distribution or distribution by community health workers may help curb misuse; however, this should be weighed against the financial costs of using such a system.

### Theme 7: Sustainability of the intervention beyond donor funding

The intervention relied on external donor funding. When the funding ended, the intervention too ceased. This meant that reliance on only external funding cannot sustain the intervention in the long term. The community leaders suggested that mobilising community contributions through different participatory mechanisms can help sustain such interventions in the long term. A community leader from one of the villages that benefited from the intervention suggested that:

I would suggest that you continue with this project because women here are ready to contribute support, and I have never heard of any woman these days saying that let me abort because I will not get support. So you continue to help them. (In Depth interview of male council representative in Iringa village, Buyende District)

Therefore, to sustain the gains achieved by the intervention, two other programmes were developed. One programme, The Maternal and Newborn Study (MANEST), is aiming at targeting the vouchers according to distance so that it is less costly while the second, Maternal and Newborn Implementation of Equitable Systems (MANIFEST), is looking at identifying local systems that can generate financial resources and systems that can be used to manage them. Such interventions should not end when funding ends; they should be developed further to make them sustainable in the local context.

### Theme 8: Unintended consequences

There is evidence that suggests that voucher schemes may encourage increased fertility if the number of children does not limit entry into the scheme [14]. In this particular project, all pregnant women benefited regardless of the number of pregnancies or children they had. Anecdotal evidence suggested that in a few cases, it may have contributed to changes in fertility decisions. During the focus group discussions, it was reported that some women decided to change their fertility decisions and to have more children because of the support provided by the programme. This is demonstrated in the quotation below:

I am grateful about this benefits we have got from this project. Continue providing us with the same good services. One has to just call a boda boda cyclist, and he will come and take you to the hospital very fast to get treatment. Even those who were on family planning have now stopped and are now conceiving because there is now free transport to hospital. (FGD of women from Nkondo village, Buyende District)

We should think about how to avoid this happening. This could be through careful detection and providing health education about family planning services plus partnering with other programmes that provide family planning services.

### Limitations

It is important to note that the effectiveness of the different methods in communicating the programme messages was not comprehensively evaluated in this study. The effect of vouchers on changes in fertility decisions needs to be studied too. The experience of mothers who were beneficiaries when 18 years and below was not captured as there was no data. Since we used secondary data from the interviews, some information could have been lost during the analysis process which involved merging similar data under themes. However we reviewed the project report and field reports to verify the findings and minimise loss of important learning. This study had no control on the selection of the participants for the interviews and how they responded. But the interviews of different groups from the participating districts were analysed, and findings from the interviews were collaborated by a review of study field reports. We believe this minimised any response bias.

## Conclusions

This study has provided key lessons for voucher schemes. Several authors have recommended that demand and supply side initiatives should be implemented together. Indeed, the results showed that the health system must be in position to respond to the demand created. Inability of the health system to meet this demand can lead to failure of the voucher project. It also showed that there is an opportunity to explore more partnerships with the private sector in order to increase utilisation of maternal and child health services. The use of private transporters led to the emergence of effective agents of change for maternity services utilisation at the health facilities. It also emphasised the importance of setting up secure systems to deal with fraud which may be a challenge in countries where corruption is high. Serious consideration must be given to developing a strong fraud proof system. Changes were made during the programme implementation in response to feedback from stakeholders, highlighting the importance of designs which allow modification of the intervention.

This work also emphasised the importance of iterative design and implementation, focused on identifying unintended consequences and planning for their mitigation. For example, to ensure that voucher programmes do not lead to increased fertility, they may require further partnerships with other programmes such as family planning programmes. This can create awareness about the importance and use of family planning services.

Lastly, the issue of sustainability of such projects is also important. If sustainability plans are not built into the project, benefits from such programmes may be lost at the end of the intervention. Health policy planners can therefore use this information to develop follow up programmes to test modified versions that are more sustainable. Such programmes could use locally existing community structures for management and resource mobilisation for self-sustainment.
